# Molecular Characterization of *Plasmodium* Species to Strengthen Malaria Surveillance in Migrant Populations in Honduras

**DOI:** 10.3390/tropicalmed10100292

**Published:** 2025-10-15

**Authors:** Ashley Godoy, Kevin Euceda, Alejandra Pinto, Hugo O. Valdivia, Lesly Chaver, Gloria Ardon, Gustavo Fontecha

**Affiliations:** 1 Instituto de Investigaciones en Microbiología, Facultad de Ciencias, Universidad Nacional Autónoma de Honduras, Tegucigalpa 11101, Honduras; ashmel1417@gmail.com (A.G.); gabrielej336@gmail.com (K.E.); mpinto@unah.edu.hn (A.P.); 2 U.S. Naval Medical Research Unit SOUTH (NAMRU SOUTH), Department of Parasitology, Lima 07006, Peru; hugo.o.valdivia.ln@health.mil; 3 Laboratorio Nacional de Vigilancia de Malaria, Secretaría de Salud de Honduras, Tegucigalpa 11101, Honduras; leslychaver@yahoo.com (L.C.); manaardon@gmail.com (G.A.)

**Keywords:** migration, malaria, Honduras, molecular markers

## Abstract

As Honduras approaches malaria elimination, imported infections pose a growing challenge to disease surveillance and control. In this study, we analyzed 14 molecular markers—six from *Plasmodium falciparum* and eight from *P. vivax*—in samples from local and migrant subjects to assess their utility in differentiating local versus imported infections. All *P. falciparum* isolates carried the wild-type *pfcrt* haplotype associated with chloroquine susceptibility. However, polymorphisms in *pfmdr1*, *pfama1*, *pfglurp*, and *pfs47* revealed distinct genotypes in migrant versus local samples, suggesting external origins. For *P. vivax*, three novel *pvcsp* VK210 haplotypes and the first detection of a VK247 variant in Honduras were identified in migrants. Additional novel haplotypes were found in *pvmsp1*, *pvmsp3α*, *pvmsp3β*, *pvs47*, and *pvs48*/45. Several of these markers—particularly *pfmdr1*, *pfs47*, *pvs47*, and *pvs48*/45—proved informative for inferring geographic origin. This study demonstrates the value of molecular surveillance in low-transmission settings, supporting public health efforts by identifying potentially imported cases.

## 1. Introduction

Malaria remains a significant public health challenge in the Americas, although the region has achieved notable reductions in transmission over the past two decades [1]. Honduras is one of the countries in Central America where malaria continues to be endemic, primarily caused by *Plasmodium vivax*, with less than 10% of cases of *P. falciparum*. In recent years, the country has experienced a marked decline in malaria incidence, accompanied by a narrowing of the geographic range and a reduction in parasite genetic diversity [2,3]. These changes have important implications for malaria control and elimination strategies, including surveillance, diagnostics, and treatment policies.

At the same time, increasing human mobility, particularly among individuals transiting through Honduras toward North America, poses new challenges for malaria surveillance [4,5]. Individuals traveling from high-transmission areas in Africa, Asia, and South America may carry genetically distinct parasite strains into regions with limited transmission, such as Honduras [6]. The introduction of non-native *Plasmodium* strains has potential consequences for drug resistance, diagnostic effectiveness, and transmission dynamics. Thus, there is a need to distinguish between locally acquired infections and those imported from other regions, especially in the context of elimination efforts [7].

Conventional epidemiological surveillance in Honduras is led by the Ministry of Health through standardized protocols, where thick blood smear microscopy remains the gold standard for malaria diagnosis and case confirmation at the national level [8]. National surveillance manuals and operational guidelines emphasize case detection, treatment, and reporting through the health information system. While microscopy is reliable and cost-effective, it has limitations in detecting low-density and mixed infections. In this context, molecular approaches can complement conventional surveillance by improving detection sensitivity and providing species-level identification, which is particularly relevant in migrant populations that may introduce *Plasmodium* species not commonly found in Honduras.

Molecular tools have become essential in characterizing malaria parasite populations and tracking transmission dynamics. Several genetic markers, including drug resistance genes [9,10], surface antigens [3,11,12], and vaccine candidates [13,14,15,16], have been used to infer parasite origin, assess population structure, and detect novel variants. In settings with limited access to next-generation sequencing (NGS) technologies [17], targeted sequencing of informative loci offers a cost-effective alternative for genetic surveillance. In Honduras, these molecular markers have been employed for over two decades [2,3,15,18,19,20,21,22,23,24,25,26], providing a valuable baseline for comparative genetic analyses.

Therefore, the objective of this study was to evaluate the utility of 14 molecular markers—six for *P. falciparum* and eight for *P. vivax*—to distinguish between local and potentially imported malaria infections. We analyzed samples collected from both local patients and migrant individuals transiting through Honduras. By comparing these sequences to existing national and international databases, we sought to determine the informativeness of each marker in inferring geographic origin. Additionally, the study contributes to building a reference framework of parasite genetic diversity within Honduras, which may support future efforts to differentiate between relapses and reinfections in patients treated with radical cure therapies.

## 2. Materials and Methods

### 2.1. Sample Collection

A total of 238 blood samples impregnated on Whatman No. 3 filter paper, previously diagnosed with malaria by the Honduran Ministry of Health, were analyzed. These samples, collected between 2023 and 2024, were evaluated as part of quality control for microscopy or rapid diagnostic test (RDT) malaria diagnosis using molecular techniques, as well as for the analysis of drug resistance-associated genes, in compliance with national regulations and routine disease surveillance [8]. According to national guidelines, malaria cases were defined as confirmed when parasites were detected by thick blood smear microscopy or RDT. Imported cases were defined as malaria infections diagnosed in individuals with a travel history to endemic countries outside Honduras during the 30 days preceding diagnosis. Both local and migrant patients were treated according to the national treatment scheme: chloroquine for three days plus primaquine for 14 days for *P. vivax*, and chloroquine for three days plus a single dose of primaquine for *P. falciparum*. However, treatment outcomes were not available, as this information was not systematically collected for the patients included in this study. Among the total samples, five were identified as originating from patients in the migration process through Honduras to North America; these five represent the entirety of imported malaria cases detected during the study period. The samples were anonymized, but limited demographic data were available for the patients. Without available NGS capabilities, these five samples were thoroughly characterized in this study using a panel of 14 molecular markers available in our laboratory. Also, four to seven local samples were analyzed using the same panel of molecular markers. This characterization aimed to assess the level of information each marker could provide to determine whether malaria was locally acquired or originated in another geographic region. The study was reviewed and approved by the ethics committee (CEI-MEIZ) of the National Autonomous University of Honduras (UNAH) under protocol number PI 12-2024.

### 2.2. DNA Extraction and Molecular Diagnosis

DNA extraction was performed using two 2 mm diameter circles that were punched from blood-impregnated filter paper. Genomic DNA was isolated using the Extracta^®^ DNA Prep for PCR kit (QuantaBio, Beverly, MA, USA) according to the manufacturer’s protocol and stored at −20 °C until analysis. *Plasmodium* genus detection was performed using PET-PCR with primers described in Table 1 [25,27]. Samples testing positive were further amplified with species-specific primers for *P. falciparum* and *P. vivax*, the two malaria parasites endemic to Honduras. PCR reactions were prepared in a 20 μL volume containing 10 μL GoTaq^®^ Probe qPCR Master Mix (Promega Corp., Madison, WI, USA), 0.5 μL each of forward and reverse primers (10 μM) (Table 1), 4 μL nuclease-free water, and 5 μL DNA template (~40 ng/μL). Amplification was carried out on a Mic qPCR Cycler (Bio Molecular Systems, Brisbane, Australia) under the following conditions. Initial denaturation: 95 °C for 15 min, 45 cycles of 95 °C for 20 s, 63 °C for 40 s, 72 °C for 30 s. Fluorescence detection used 6FAM-labeled primers for genus identification and HEX-labeled primers for species discrimination. All runs included positive and negative controls, with a cycle threshold (Ct) ≤ 42 defined as a positive result. Data were analyzed using Mic qPCR Cycler Software v2.10.1.3.

### 2.3. Molecular Markers for P. falciparum: pfcrt, pfmdr1, pfglurp, pfama1, pfhrp2/3, and pfs47

In total, one *P. falciparum* sample (M1) and four *P. vivax* samples (M2–M5) were analyzed using the molecular markers listed in Table 2 and Table 3, with the country of origin indicated for each sample.

One sample (designated “M1”) obtained from a foreign subject with falciparum malaria migrating through the country was analyzed. The subject was recruited in the Cortés department, though their country of origin and most recent geographic provenance remain unknown. Additionally, four samples from local patients were analyzed. A 264 bp region of *pfcrt* exon 2 (encoding amino acids 72–76), known to harbor polymorphisms linked to *P. falciparum* drug resistance, was amplified and sequenced using nested PCR (nPCR) with primers described in Table 1 [3,29]. Amplicons were visualized on 1% ethidium bromide-stained agarose gels, purified, and sequenced by Psomagen Inc. (Rockville, MD, USA) using primer AL5631 (Table 1). Sequences were analyzed in Geneious Prime v.2024.05 to assess residues 72–76 of *pfcrt*.

Three regions of the *pfmdr1* gene were amplified to identify five polymorphisms at codons 86 and 184 (first segment), 1034 and 1046 (second segment), and 1246 (third segment). The three regions were amplified by nested PCRs according to a previous report [20] (Table 1). PCR products were resolved on 2% agarose gels stained with ethidium bromide and visualized under UV transillumination. The products were sequenced from both sides.

A semi-nested and a nested PCR were used to amplify a fragment of the *P. falciparum glurp* [3,11] and *ama1* genes [3,37], respectively (Table 1). Amplicons were bidirectionally sequenced and subsequently aligned to generate a consensus sequence for each individual. The nucleotide sequences of *pfglurp* and *pfama1* were translated into amino acid sequences using the correct open reading frame (ORF). The identified haplotypes were compared against those previously reported as circulating in the country. For any novel haplotypes discovered, sequences were deposited in GenBank, and accession numbers were assigned.

The presence or absence of partial coding regions between exons 1 and 2 of the *pfhrp2* and *pfhrp3* genes was assessed using two nested PCR assays as previously described [18,19] (Table 1). Positive and negative controls were used within each experiment. For the positive controls, we used DNA from previously confirmed clinical isolates available in our laboratory, which had been validated in earlier published studies and samples confirmed by sequencing in prior work. All negative samples were screened a second time and samples showing double deletions in both target regions underwent a third round of amplification.

Putative SNPs at positions 707/718/725 of the polymorphic region of domain 2 of the *pfs47* gene were genotyped as previously described [15,31] (Table 1). Sample “M1” from the migrant and two local samples were sequenced.

### 2.4. Molecular Markers for P. vivax: pvcsp, pvmsp1-F2 block, pvmsp3α, pvmsp3β, pvs47, and pvs48/45

Four samples obtained from foreign patients with vivax malaria migrating through the country were analyzed. The subjects were recruited in the Cortés and El Paraíso departments. One of these subjects was from Afghanistan (“M2”), one from Venezuela (sample designated “M3”), and the nationality of the other two was unknown (“M4” and “M5”). Several local samples were also analyzed.

Seven protein-coding genes expressed on the surface of different *P. vivax* life stages were amplified and bidirectionally sequenced to assess sequence polymorphisms. For *pvcsp*, sequencing enabled classification between the two major types (VK210 and VK247). The *pvcsp* and *pvmsp1* (F2) genes were amplified and sequenced as previously described [24], employing the primers detailed in Table 1. Similarly, the *pvmsp3α* and *pvmsp3β* genes were analyzed [3]. Nucleotide sequences were translated to amino acid sequences using the correct ORF, and identified haplotypes were compared against those previously reported as circulating in the country. Novel haplotypes were deposited in GenBank, with corresponding accession numbers assigned.

The full coding sequences of two genes encoding antigens proposed as potential transmission-blocking vaccine (TBV) candidates—*Pvs47* and *Pvs48/45*—were amplified and sequenced using primers previously described [16,34,35,36].

*Pvs47* was amplified by PCR in a 50 µL reaction volume containing 25 µL of 2X Taq Master Mix (Promega Corp., Madison, WI, USA), 2 µL of each primer at 10 µM (Table 1), 2 µL of BSA (10 mg/mL), and 4 µL of genomic DNA. The PCR cycling conditions consisted of an initial denaturation at 95 °C for 5 min, followed by 25 cycles of 95 °C for 1 min, 58 °C for 1 min, and 72 °C for 2 min, with a final extension at 72 °C for 10 min. A nested PCR (nPCR) was performed using 1 µL of the first-round PCR product in a 50 µL reaction containing 25 µL of Taq Master Mix, 2 µL of each nested primer at 10 µM (Table 1), and 2 µL of BSA (10 mg/mL). The program for the nested PCR included an initial denaturation at 95 °C for 5 min, followed by 35 cycles at 95 °C for 1 min, 58 °C for 1 min, and 72 °C for 2 min, with a final extension at 72 °C for 10 min. Amplicons were visualized by agarose gel electrophoresis, stained with ethidium bromide, and sequenced bidirectionally using the internal primers. A total of 23 samples from local patients and 3 from foreign migrant patients were successfully sequenced. All sequences were aligned and analyzed for the presence of polymorphisms.

The *pvs48*/45 gene was also amplified using several PCR reactions and under the same conditions described for *pvs47* except that the annealing temperature of the second PCR was 53 °C. Primers used in the first and second reactions are listed in Table 1. Given that the gene is over 1350 nucleotides in length, its full sequence could not be obtained using only the primers from the second semi-nested PCR reaction. Therefore, nine internal primers were employed to assemble each complete sequence (Table 1, Sequencing primers). A total of 23 sequences from local isolates and 3 from migrant patients were sequenced and assembled. Sequences obtained were deposited in GenBank, with corresponding accession numbers assigned. For both *pvs47* and *pvs48*/45, homologous sequences were deposited in GenBank and accession numbers were assigned.

## 3. Results

### 3.1. Molecular Markers for P. falciparum

Genotype and phenotype analysis was performed for two parasite genes associated with antimalarial drug resistance, *pfcrt* and *pfmdr1*. In this context, the term “phenotype” refers to the in silico translation of the gene sequences into their corresponding amino acid sequences, which allows the prediction of potential resistance-associated profiles. The *pfcrt* wild-type haplotype (72CVMNK76), indicating chloroquine susceptibility, was detected in all samples (*n* = 4), including the sample of the migrant subject named “M1”. *pfmdr1* polymorphisms differed between the migrant “M1” (N86/184F/S1034/N1042/D1246) and local (N86/184F/1034C/1042D/D1246) cases, suggesting distinct genetic profiles (Table 2).

Additionally, partial coding regions of two genetic diversity markers (*pfglurp* and *pfama1*) were sequenced. For *pfglurp*, the migrant patient’s sample “M1” revealed a novel haplotype not previously reported in Honduras, differing by two residues from the reference sequence (GenBank accession PP681141). This new sequence was deposited in GenBank under accession PV567568. The remaining three local sequences were identical to the reference sequence PP681141. Similarly, the “M1” sample revealed a novel *pfama1* haplotype, not previously reported in the country, which differed by 7 residues from the reference sequence (GenBank accession PP795732). This new haplotype was deposited in GenBank under accession number PV567569. Local samples’ sequences were identical to PP795732 (Table 2).

We also assessed the presence/absence of *pfhrp2* and *pfhrp3*, genes encoding antigens widely used in malaria rapid diagnostic tests (RDTs). The migrant subject’s sample tested positive for both loci (*pfhrp2*+/*pfhrp3*+), whereas local samples exhibited an *pfhrp2*+/*pfhrp3*− genotype (Table 2).

Regarding *pfs47*, three polymorphic positions of domain 2 of the *pfs47* gene (707, 718, and 725), which have been used to infer the possible geographic origin of parasite strains [31], were evaluated. “M1” exhibited the 707C/718C/725C genotype, whereas the local samples showed the T707/T718/T725 genotype (Table 2).

### 3.2. Molecular Markers for P. vivax

**Figure 1:**
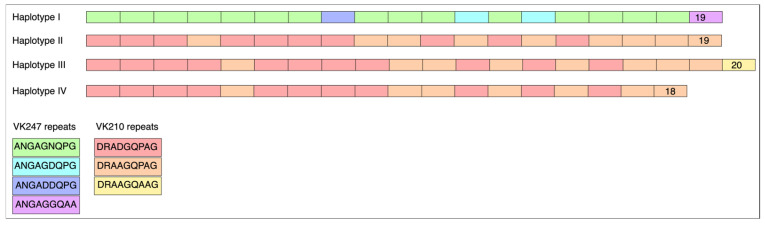
Schematic representation of the amino acid motifs of the three allelic *pvcsp* variants bearing the VK210 repeat type and one bearing the VK247 type.

We evaluated six molecular markers (*pvcsp*, *pvmsp1*-F2 block, *pvmsp3αN1* and *N2*, and *pvmsp3βN1* and *N2*) commonly used to assess *P. vivax* genetic diversity. For *pvcsp*, all analyzed samples—including two from migrant individuals—exhibited four VK210-type allelic variants (“M4” and “M5”), three of which were novel haplotypes and previously unreported in Honduras (Figure 1). These new variants were deposited in GenBank (Accession No. PV567570-2) (Table 3). Notably, one sample from an Afghan subject (“M2”) revealed a VK247-type variant, which constitutes the first report of this genotype in Honduras (Accession No. PV567574).

We additionally sequenced the F2 segment of *pvmsp1*, encompassing one conserved block and two variable blocks (blocks 6 and 8) of the gene. Two samples collected from migrant patients (“M3” and “M5”) exhibited haplotypes previously reported in Honduras (GenBank Accession No. JQ903607 and JQ903606). In comparison, two local patient samples revealed novel haplotypes not previously described in the country (Acc. No. PV590043 and PV594050) (Table 3).

The two remaining genetic markers, *pvmsp3α* and *pvmsp3β*, were sequenced in both directions. Consensus sequences could not be generated due to the length of the sequences and the difficulty in aligning the forward primer-derived sequences with those obtained from the reverse primer. Consequently, each sequence was considered as an individual marker (*pvmsp3α-N1*, *pvmsp3α-N2*, *pvmsp3β-N1*, and *pvmsp3β-N2*). Regarding the *pvmsp3α*-*N1* locus, two samples (“M3” and “M5”) exhibited a haplotype previously reported in the country (GenBank accession numbers PP913947-8 and PP795720). A third sample from a migrant subject (“M4”) revealed a novel haplotype, not previously documented in Honduras, which was deposited in GenBank under accession number PV590041. This haplotype showed 100% similarity with sequences reported in the Americas (e.g., AAO20877, AGR50762) and Asia (e.g., WQM98282). For the *pvmsp3β*-*N1* locus, all three migrant samples displayed the same haplotype, which had not been previously reported in the country (Acc. No. PV590042). One local sample also revealed a novel haplotype (Acc. No. PV594049). Sequencing with the N2 primer (for both *pvmsp3α* and *pvmsp3β*) did not reveal any new haplotypes in Honduras. The three migrant patient samples all exhibited known haplotypes: *pvmsp3α* (Acc. No. PP913961-2) and *pvmsp3β* (Acc. No. PP795728-31, PP886070) (Table 3).

#### *pvs47* and *pvs48/45*

The coding sequence of the *pvs47* gene, comprising 1299 nucleotides, was sequenced in 23 samples from local patients and 4 from migrant subjects. Translation of this sequence yielded a 433-residue polypeptide. A single SNP was detected at position 22 of the polypeptide (F22L). Seven samples exhibited the F22 phenotype, two of which were from migrant individuals (“M2” and “M4”), while the remaining samples displayed the 22L phenotype, including “M3” and “M5”. Table 3 also describes 13 additional positions that have been reported as polymorphic in the literature [14,16,35]. GenBank accession numbers were assigned to the genotypes described in this study (Acc. No. PV700528-9, PV30102).

The *pvs48*/*45* gene, consisting of 1350 nucleotides and encoding 450 amino acids, was also sequenced in 23 local samples and 3 from migrant individuals. Three polymorphic positions were identified: H211N, K250N, and E353Q. “M2” exhibited the H211/250N/353Q phenotype, while “M3” and “M5” revealed the 211N/K250/E353 genotype. “M4” could not be successfully sequenced. An additional nine positions previously reported as polymorphic [16] are shown in Table 3. The remaining local sequences (*n* = 18) revealed the haplotype E35/Y196/K211/K250/D335/E353/A376/I380/K390/K418 (Acc. No. PV700525-7).

## 4. Discussion

Human mobility represents one of the main challenges for infectious disease control globally. Migratory flows can facilitate the introduction of vector-borne diseases, including malaria, into regions with low endemicity or near elimination [38]. Such mobility poses the additional risk of introducing *Plasmodium* strains carrying drug resistance mutations or genetic variants not previously detected locally. In this context, the implementation of molecular tools for malaria surveillance is increasingly relevant, as they provide valuable information that complements conventional case surveillance and supports regional elimination goals [39].

In Honduras, malaria surveillance is coordinated by the Ministry of Health through standardized protocols in which thick blood smear microscopy remains the gold standard for case confirmation and reporting [8]. All confirmed malaria cases are entered into the national epidemiological information system, which serves as the country’s repository of case data. While microscopy is reliable and cost-effective, its limitations in detecting low-density and mixed infections highlight the potential of molecular approaches to add resolution by identifying *Plasmodium* species and genetic variants, and by contributing to the monitoring of drug resistance [20,40]. The present study was therefore designed not to replace, but to complement conventional malaria surveillance with molecular evidence.

In this study, six molecular markers of *P. falciparum* and eight of *P*. *vivax* were analyzed in samples from malaria patients in transit through Honduras en route to northern regions of the Americas, as well as in samples from local patients. The objective of characterizing these *Plasmodium* isolates was to evaluate the informativeness of the 14 molecular markers in distinguishing whether the infections originated from external geographic regions or within the national territory. Additionally, a detailed characterization of the genotypes circulating within the country may soon become a valuable tool for distinguishing relapses from reinfections in malaria patients treated with primaquine or tafenoquine, especially in a context of limited access to next-generation sequencing (NGS) technologies [41].

The first marker evaluated in patients with *P. falciparum* malaria was *pfcrt*, a transporter gene whose mutations at codons 72 to 76 confer resistance to chloroquine (CQ). The haplotype identified in “M1”, a sample from a migrant patient, was 72CVMNK76, described as the wild-type phenotype and associated with CQ susceptibility. CQ-resistant phenotypes (CVIET, SVMNT, CVIDT, and CVMNT) have been reported for several decades in nearly all malaria-endemic regions [42], except Central America, where the wild-type CVMNK remains the only circulating haplotype [3,20,21,43]. The fact that patient “M1” was infected with a strain carrying the wild-type *pfcrt* phenotype suggests that the origin of the infection may have been Honduras or a neighboring country, including Haiti or the Dominican Republic. However, the withdrawal of CQ as treatment in several African countries has led to an almost complete re-emergence of CQ-sensitive parasite populations [9,29,44,45,46,47], along with the appearance of some CQ-sensitive populations in Asia [48]. Consequently, the CVMNK haplotype can no longer be considered exclusive to the Central American region.

A second antimalarial drug resistance marker evaluated in this study was *pfmdr1*. The haplotype detected in “M1” was N86/184F/S1034/N1042/D1246 (NFSND haplotype), which differs at positions 1034 and 1042 from the local strains, all of which exhibited the NFCDD phenotype. Previous studies have analyzed the *pfmdr1* gene in strains collected in Honduras. In 2011, the N86 phenotype was identified in 30 samples from Honduras, while one sample from Africa and one from the Pacific region showed the mutant 86Y genotype [22]. In 2020, Valdivia et al. analyzed 16 strains from Honduras that revealed the N86, 184F, D1246 haplotype [17]. In 2021, 51 local strains were analyzed, and all displayed the NFCDD haplotype [20]. On the other hand, the reference strain HB3, originally isolated in Honduras, carries the NFSDD haplotype. Thus, the NFSND haplotype had not been previously reported in Honduras and may be indicative of an infection acquired outside the national territory.

Two genetic markers widely used to estimate parasite genetic diversity were also sequenced. For both *pfglurp* and *pfama1*, “M1” exhibited haplotypes that had not been previously reported in Honduras. Recently, Zamora et al. analyzed both genes in 90 *P. falciparum* strains collected from malaria-endemic regions of Honduras [3]. In both cases, 89 out of the 90 strains were identical, and for *pfglurp*, only one strain showed a 56-nucleotide deletion, indicating high homogeneity in these markers. A GenBank search for the *pfglurp* genotype found in “M1” showed 100% identity with the 3D7 strain (Acc. No. MG578504) and with other entries reported from India and Indonesia (Acc. Nos. KY425859–64–95, AF191066), among others. For *pfama1*, the highest similarity of the genotype found in “M1” to sequences in GenBank was 98.42%. Given the low genetic diversity of the parasite population in Honduras, the detection of divergent variants in *pfglurp* and *pfama1* in “M1” further supports the hypothesis of an imported strain from a foreign country.

Our study also screened for deletions on *pfhrp2* and *pfhrp3*. Their presence or absence has been relevant since 2010 due to their involvement in rapid diagnostic tests (RDTs) [49]. The “M1” sample showed the presence of both gene segments (*pfhrp2*+/*pfhrp3*+), whereas six local samples displayed the *pfhrp2*+/*pfhrp3*− haplotype. Deletions at both loci have been previously investigated in Central America. The first study, published in 2015, analyzed 68 samples collected in the city of Puerto Lempira. All samples tested positive for *pfhrp2*, and only 50% were positive for *pfhrp3* [18]. In 2018, a follow-up study was published analyzing 128 samples from Honduras, Guatemala, and Nicaragua [19]. In that study, only 8.6% of the isolates were *pfhrp3*+, suggesting that the *pfhrp2*+/*pfhrp3*+ haplotype is not common in Honduras, although its occurrence in the circulating parasite population cannot be entirely ruled out.

The final *P. falciparum* marker analyzed was *pfs47*, a gene that mediates the parasite’s ability to evade the mosquito immune system and is therefore critical for parasite survival and transmission [50]. It has been shown that *pfs47* exhibits strong geographic population structure, with distinct haplotypes predominating across continents [31,41]. The major differences among these haplotypes are based on polymorphisms at nucleotide positions 707, 718, 725, and 742. Sequencing of the gene in sample “M1” revealed the genotype 707C/725C (CC), whereas local samples in this study and 31 additional samples analyzed in a previous study [15] displayed the 707T/725T (TT) genotype. The TT genotype has been described as the most frequent in Latin America, while the CC genotype is more common in Africa. This evidence supports the hypothesis of an infection acquired outside the country.

We also analyzed four molecular markers commonly used to assess *P. vivax* genetic diversity—*pvcsp*, *pvmsp1* (F2 block), *pvmsp3α*, and *pvmsp3β*—in both local and migrant patient samples. All *pvcsp* sequences belonged to the VK210 type, except one from an Afghan migrant (“M2”), which revealed the VK247 genotype, marking its first report in Honduras, supporting the hypothesis that this isolate may have a non-American origin. Among the VK210 variants, three novel haplotypes were identified compared to those previously described in a study that sequenced 84 samples collected from all malaria-endemic regions at that time [24]. Sequencing of the *pvmsp1*-F2 segment revealed two migrant samples (“M3” and “M5”) with known Honduran haplotypes, while two local samples exhibited previously undescribed variants [24], suggesting locally acquired infections. Unfortunately, *pvmsp1* could not be sequenced in “M2” or “M4”.

At the *pvmsp3α*-*N1* locus, two migrant samples carried known haplotypes [3], while a third (“M4”) displayed a novel variant with 100% similarity to sequences from South America (Acc. No. AAO20877, AGR50762) and Myanmar (Acc. No. WQM98282). This result, along with the novel VK210 haplotype found for *pvcsp*, would also suggest that this parasite strain could have been imported. For *pvmsp3β*-*N1*, all three migrant samples and one local sample revealed previously unreported haplotypes [3]. Thus, this locus does not allow for discrimination of the parasites’ origin.

Finally, the complete coding sequences of two candidate transmission-blocking vaccine genes, *pvs47* and *pvs48*/45, were sequenced. In *pvs47*, only a single SNP was detected, resulting in a phenylalanine at residue 22 in samples “M2” and “M4,” whereas local samples and the other two migrant samples displayed the 22L phenotype. For *pvs48*/*45*, three polymorphic positions were identified: H211N, K250N, and E353Q. Sample “M2” exhibited the H211/250N/353Q phenotype, while “M3” and “M5” showed the 211N/K250/E353 genotype. “M4” could not be successfully sequenced. Eighteen local samples revealed a diverse haplotype (K211/K250/E353). These findings strongly suggest that both markers are highly informative for inferring the geographic origin of *P. vivax* strains, particularly in the current context of low transmission and reduced genetic diversity in Honduras.

Taken together, these results demonstrate the value of incorporating molecular markers as a complementary tool to the national malaria surveillance system. By situating the findings in the broader problem of imported diseases, we emphasize that molecular approaches add resolution to case detection, help identify imported strains with potential drug resistance, and provide data on parasite population structure. These contributions are particularly relevant in countries like Honduras, where malaria transmission is now focalized, and where elimination goals will depend on distinguishing local from imported cases with greater accuracy.

## 5. Conclusions

This study fulfilled its objective of assessing the utility of multiple molecular markers to differentiate between locally acquired and potentially imported malaria infections in Honduras, a country currently experiencing low transmission and limited genetic diversity of the parasites. By analyzing six *P. falciparum* and eight *P. vivax* genetic markers, we identified novel haplotypes and allelic variants that had not been previously reported in the country, particularly in samples from migrant patients. Some markers—such as *pfmdr1*, *pfama1*, *pfs47* for *P. falciparum*, and *pvcsp*, *pvmsp3α*, and the transmission-blocking vaccine candidates *pvs47* and *pvs48/45* for *P. vivax*—proved especially informative in inferring the likely geographic origin of infections. These findings demonstrate the value of molecular surveillance in detecting the introduction of foreign strains and complementing conventional surveillance systems and in guiding public health strategies, especially in settings where advanced sequencing technologies remain unavailable. We also acknowledge the main limitations of the study, including the small number of migrant samples and the lack of systematic follow-up of clinical outcomes, which should be addressed in future research with larger cohorts.

## Figures and Tables

**Table 1:** List of primers used for the amplification of molecular markers of *Plasmodium vivax* and *P. falciparum.*

Target Gene	Reaction	Primer Name	Primer Sequence (5′-3′)	References
18Sr RNA gene	PET-PCR for genus *Plasmodium*	Genus forward	GGC CTA ACA TGG CTA TGA CG	[25,27]
		Genus reverse	6FAM-agg cgc ata gcg cct gg CTG CCT TCC TTA GAT GTG GTA GCT	
18Sr RNA gene	PET-PCR for *P. falciparum*	Falciparum forward	ACC CCT CGC CTG GTG TTT TT	[27]
		Falciparum reverse	HEX-agg cgc ata gcg cct gg TCG GGC CCC AAA AAT AGG AA	
18Sr RNA gene	PET-PCR for *P. vivax*	Vivax forward	ACT GAC ACT GAT GAT TTA GAA CCC ATT T	[28]
		Vivax reverse	HEX- agg cgc ata gcg cct ggT GGA GAG ATC TTT CCA TCC TAA ACC T	
*pfcrt*	1st round	AL6821	AGC AAA AAT GAC GAG CGT TAT AG	[3,29]
		AL6822	ATT GGT AGG TGG AAT AGA TTC TC	
	2nd round	AL5631	TTT TTC CCT TGT CGA CCT TAA C	
		AL5632	AGG AAT AAA CAA TAA AGA ACA TAA TCA TAC	
*pfmdr1*	SNPs 86, 184	MDR1-1F	TTA AAT GTT TAC CTG CAC AAC ATA GAA AAT T	[20]
		MDR1-1R	CTC CAC AAT AAC TTG CAA CAG TTC TTA	
		MDR1-2F	TGT ATG TGC TGT ATT ATC AGGA	
		MDR1-2R	CTC TTC TAT AAT GGA CAT GGTA	
*pfmdr1*	SNPs 1034, 1046	1042-A	GTC GAA AAG ACT ATG AAA CGT AGA	[20]
		1042-C	CTC AAA TGA TAA TTT TGC AT	
		1042-B	GAT CCA AGT TTT TTA ATA CA	
		1042-C	CTC AAA TGA TAA TTT TGC AT	
*pfmdr1*	SNP 1246	1246-A	GTG GAA AAT CAA CTT TTA TGA	[20]
		1246-B	TTA GGT TCT CTT AAT AAT GCT	
		1246-C	GAC TTG AAA AAT GAT CAC ATT	
		1246-D	GTC CAC CTG ATA TGC TTT T	
*pfglurp*	1st round	GLURPOF	TGA ATT TGA AGA TGT TCA CAC TGA AC	[3,11]
		GLURPOR	GTG GAA TTG CTT TTT CTT CAA CAC TAA	
	2nd round	GLURPGNF	TGT TCA CAC TGA ACA ATT AGA TTT AGA TCA	
		GLURPOR	GTG GAA TTG CTT TTT CTT CAA CAC TAA	
*pfama1*	1st round	Pfama1F	GTA CTT GTT ATA AAT TGT ACA	[30]
		Pfama1R	TTT TAG CAT AAA AGA GAA GC	
	2nd round	Pfama1F1	ACA AAA ATG AGA AAA TTA TAC TGC	[30]
		Pfama1R1	TTA ATA GTA TGG TTT TTC CAT CAG AAC	
*pfhrp2* exons 1–2	1st round	2E12F1	GGT TTC CTT CTC AAA AAA TAA AG	[18,19]
		2E12R1	TCT ACA TGT GCT TGA GTT TCG	
	2nd round	2E12F	GTA TTA TCC GCT GCC GTT TTT GCC	
		2E12R	CTA CAC AAG TTA TTA TTA AAT GCG GAA	
*pfhrp3* exons 1–2	1st round	3E12F1	GGT TTC CTT CTC AAA AAA TAA AA	[18,19]
		3E12R1	CCT GCA TGT GCT TGA CTT TA	
	2nd round	3E12F	ATA TTA TCG CTG CCG TTT TTG CT	
		3E12R	CTA AAC AAG TTA TTG TTA AAT TCG GAG	
*pfs47*		Pfs47_SNP707_F	GAA GAA ACT ATT GTA GAA TCT GGA AA	[15,31]
		Pfs47SNP725_R	AAG GCA TTT TTA TAA CCA CAT TAT TA	
*pvcsp*	1st round	VCS-OF	ATG TAG ATC TGT CCA AGG CCA TAA A	[24,32]
		VCS-OR	TAA TTG AAT AAT GCT AGG ACT AAC AAT ATG	
	2nd round	VCS-NF	GCA GAA CCA AAA AAT CCA CGT GAA AAT AAG	
		VCS-NR	CCA ACG GTA GCT CTA ACT TTA TCT AGG TAT	[24,33]
*pvmsp1* F2 block	1st round	VMI-O2F	GAT GGA AAG CAA CCG AAG AAG GGA AT	[24,32]
		VMI-O2R	AGC TTG TAC TTT CCA TAG TGG TCC AG	
	2nd round	VMI-N2F	AAA ATC GAG AGC ATG ATC GCC ACT GAG AAG	
		VMI-O2R	AGC TTG TAC TTT CCA TAG TGG TCC AG	
*pvmsp3α*	1st round	Pvmsp3a P1	CAG CAG ACA CCA TTT AAG G	[3,12]
		Pvmsp3a P2	CCG TTT GTT GAT TAG TTG C	
	2nd round	Pvmsp3a N1	GAC CAG TGT GAT ACC ATT AAC C	
*pvmsp3β*	1st round	Pvmsp3b P1	GTA TTC TTC GCA ACA CTC	
		Pvmsp3b P2	CTT CTG ATG TTA TTT CCA G	
	2nd round	Pvmsp3b N1	CGA GGG GCG AAA TTG TAA ACC	
		Pvmsp3b N2	GCT GCT TCT TTT GCA AAG G	
*pvs47*	1st round	CM 000453 Fwd	CAC ACC ACC GCA AAC AGG	[16]
		CM 000453 Rev	GTG CAC ATT CCG CGG TTG	
	2nd round	nst 1440 Fwd	GCG GTC CAC CCT AAC TGT AA	This study
		nst 1440 Rev	TGC TGC AAA CCA CAC ATG T	[34]
	Sequencing primers	pPvs47-F	ATA TTT CCA ACG AAG CAT TTA TGC	
		pPvs47-R	TTT TCC ATT ATG CTC ACA AAC GC	
		Pvs47F2	GAA GAA AGG GGA GGA CCA AG	[16]
*pvs48*/45	1st round	Pvs4845F	GGA ATA ATT TCG ACC ACT C	[35]
		887	TCA GAA GTA CAA CAG GAG GAG CAC	[36]
	2nd round	866	ATG TTG AAG CGC CAG CTC GCC AA	
		887	TCA GAA GTA CAA CAG GAG GAG CAC	
*pvs48*/45	Sequencing primers	pPvs4845F	ATG GCC AAA GGA GAG GTC AAG TAC	[34]
		pPvs4845R	TCG GCA GAT GCA AGT GAA GGA AGT C	
		pPvs48/45-6C-F	TCG GCA GAT GCA AGT GAA GGA AGT C	
		Pvs4845FF	TGT AAA ATC TGC GGA CGT GA	[16]
		Pvs4845RR	CGGGTGCTTTAAAAATGGAA	
		1001F	GAATGAGTTGCCCTGGGGAA	This study
		1025F	TGCCCGAGTGCTTCTTTCAA	
		249R	TCCTGGGATCTTCTTCGGGA	
		494R	AAGGCCACTCTTCCCTTCAC	

**Table 2:** Results of *P. falciparum* molecular markers applied to sample “M1”.

Sample	Origin	*pfcrt*	*pfmdr1*	*pfhrp2*	*pfhrp3*	*pfama1*	*pfs47*
M1	Unknown	72CVMNK76	NFSND	Present	Present	New haplotype (Acc. No. PV567569)	707C, 725C

**Table 3:** Results of *P. vivax* molecular markers applied to samples “M2” to “M5”.

Sample	Origin	*pvcsp*	*pvmsp3a*-*N1*	*pvmsp3a*-*N2*	*pvmsp3b*-*N1*	*pvmsp3b*-*N2*	*pvmsp1*(F2)	*pvs47*	*pvs48*/*45*
M2	Afghanistan	VK247 type. New haplotype (Acc. No. PV567574)	-	PD *	New haplotype (Acc. No. PV590042)	PD	-	F22, F24, K27, D31, S57, S62, L82, D156, V230, M233, E240, I262, I273, A373	E35, Y196, H211, K250, D335, E353, A376, I380, K390, K418
M3	Venezuela	-	PD	PD	New haplotype (Acc. No. PV590042)	PD	PD	22L, F24; K27, D31, S57, S62, L82, D156, V230, M233, E240, I262, I273, A373	E35, Y196, 211N, K250, D335, E353, A376, I380, K390, K418
M4	Unknown	VK210 type. New haplotype (Acc. No. PV567571)	New haplotype (Acc. No. PV590041)	PD	-	-	-	F22, F24; K27, D31, S57, S62, L82, D156, V230, M233, E240, I262, I273, A373	-
M5	Unknown	VK210 type. New haplotype (Acc. No. PV567570)	PD	PD	New haplotype (Acc. No. PV590042)	PD	PD	22L, F24; K27, D31, S57, S62, L82, D156, V230, M233, E240, I262, I273, A373	E35, Y196, 211N, K250, D335, E353, A376, I380, K390, K418

* PD = Previously described in the country.

## Data Availability

The original contributions presented in this study are included in the article. Further inquiries can be directed to the corresponding author. The newly generated sequences were submitted to the GenBank database under the accession numbers cited in the text.

## Abbreviations

The following abbreviations are used in this manuscript:

NGS	Next Generation Sequencing
CQ	Chloroquine
Pvcsp	*Plasmodium vivax* circumsporozoite protein
Pvmsp3a	*Plasmodium vivax* merozoite surface protein 2 alpha
Pvmsp3b	*Plasmodium vivax* merozoite surface protein 2 beta
Pvmsp1	*Plasmodium vivax* merozoite surface protein 1
Pvs47	*Plasmodium vivax* surface protein 47
Pvs48/45	*Plasmodium vivax* surface protein 48/45
Pfcrt	*Plasmodium falciparum* chloroquine transporter
Pfmdr1	*Plasmodium falciparum* multidrug resistance 1
Pfhrp2/3	*Plasmodium falciparum* histidine rich proteins 2/3
Pfama1	*Plasmodium falciparum* apical membrane antigen 1
Pfs47	*Plasmodium falciparum* surface protein 47
